# Corneal Scheimpflug topography values to distinguish between normal eyes, ocular allergy, and keratoconus in children

**DOI:** 10.1038/s41598-021-03818-3

**Published:** 2021-12-20

**Authors:** Matheus Ivan Schmitz Vieira, Alessandro Adad Jammal, Carlos Eduardo Leite Arieta, Monica Alves, Jose Paulo Cabral de Vasconcellos

**Affiliations:** Department of Ophthalmology and Otorhinolaryngology, School of Medical Sciences, University of Campinas, Rua Tessália Vieira de Camargo, Cidade Universitária, Campinas, São Paulo 13083887 Brazil

**Keywords:** Paediatric research, Corneal diseases

## Abstract

To identify and compare keratometric, corneal thickness, and elevation parameters and indices among healthy children, ocular allergy, and keratoconus using the OCULUS Pentacam Scheimpflug topography system. This study included healthy children, children with ocular allergy (OA) without keratoconus, and children with keratoconus (KC). The study design consisted of a prospective evaluation and review of medical records from a Brazilian ophthalmology department. The exclusion criteria were inability to undergo the ocular exam, other ocular diseases, contact lens wear, and topographic corneal ectasia. The effect of each corneal parameter was evaluated using univariate and multivariate logistic regression models adjusted for sex and age, and ROC curves were used to assess the ability each variable to discriminate among groups. A total of 182 subjects were included: healthy children (n = 99), children with OA (n = 32), and children with KC (n = 51). Groups differed in terms of sex, with more males in the OA group (73.2%) and the KC group (67.7%) than in the control group (40.9%). All corneal parameters studied differed significantly between the control and KC groups, and between the OA and KC groups; they also differed significantly between the three groups in terms of astigmatism, q-value, CCT, TP, BAD-D, and ARTmax values. We present the first study to describe and compare corneal tomographic parameters in healthy children, OA, and KC. Keratometry indices, ACD, ARTmax, AETP, and PETP were found to be the most useful for differentiating between healthy and KC children.

IBR registry number: CAAE 54921916.9.0000.5404.

## Introduction

Keratoconus (KC) is an ectatic corneal disease characterized by the progressive stromal thinning, protrusion of the cornea, irregular astigmatism, and vision impairment. The prevalence of KC in general population is estimated to be around 1 in 2000^1^, but it varies in different population, ethnicity, age, and studies according to different diagnose criteria, variance in genetics, environmental factors, nutrition, and diagnose tools^2^. Some studies report incidences between 5 and 23 in 10,000, and prevalence 4 and 60 out of 100,000^3^. According to the Intelligence Research in Sight Registry of American Academy of Ophthalmology, the prevalence of KC in pediatric population is 0.16% in USA^4^. The prevalence of pediatric KC Saudi Arabia is reported to be as high as 4.79%^5^.

Advanced KC can be easily diagnosed in a slit-lamp exam and based on anterior curvature measurements^6^. However, the subtle changes in the anterior corneal curvature that occur prior to the development of visual abnormalities and the typical clinical findings can be observed only using automated corneal topography methods^7^. Scheimpflug-based cameras, such as the Pentacam (Oculus, Wetzlar, Germany), Galilei (Ziemer, Biel, Switzerland), and Sirius (Costruzione Strumenti Oftalmici, Florence, Italy), are highly effective in the early detection of KC^8^. Because the Scheimpflug principle works with maximal possible depth of focus and minimal image distortion, it provides valuable information on the anterior segment of the eye^9^. Further clinical and technological advances have produced a variety of indices that allow physicians to quantify the severity of corneal irregularity^10,11^. Despite the vast amount of studies evaluating corneal parameters in adults^12^ and healthy children^13^, similar data on children with corneal irregularities are still scarce^14^. The lack of such information may limit the use of the Scheimpflug technology to identify abnormal corneas earlier in the disease’s progression.

A major risk factor for KC is ocular allergy. Ocular allergy is a blanket term to describe different types of allergies, the most commonly diagnosed of which are seasonal and perennial allergic conjunctivitis. Ocular allergy prevalence ranges from 15 to 25% of the population^15^, and is increasing in the past few decades. In Brazil, a study in 2006 revealed the prevalence of rhinoconjunctivitis at 12%, while a report in 2012 estimated the prevalence of allergic conjunctivitis at 20.7% in preschool children^16^. A number of inflammatory mediators are expressed in the cornea and ocular surface of patients with ocular allergy , including histamine, protease, tumoral necrosis factor alpha (TNF-α), interleukins (IL), and matrix metalloproteinases (MMP)^17^, and these mediators may contribute to the development and progression of KC^18–22^.

KC onset usually occurs during puberty^2,23^; however, when it develops earlier, it reaches more advanced stages sooner and progresses faster^24^, leading to greater decreases in quality of life, worsened visual acuity, and negative impacts on social and educational development^25^. Childhood KC is also associated with an elevated risk of delayed diagnosis, corneal scarring, and penetrating keratoplasty^26^. In France, a study found KC stage 4 (Amsler Krumeich classification) was diagnose in 27.8% of patients below 15 years old against 7.8% with age more than 27 years at the time of diagnosis with male predominance^24^. And Chatzis et al*.* has observed a KC progression in 88% of children by one year of diagnosis mandating an early advocation of corneal crosslinking (CXL) treatment in these pediatric eyes^27^. Comprehensive corneal evaluations are sensitive to the subtle changes in parameters in children in subclinical stages of KC and with suspicious corneal patterns; these exams may also be used to evaluate progression and establish therapeutic strategies. These factors highlight the importance of early diagnosis in children, particularly in children with ocular allergy and eye-rubbing habits.

Given the importance of keratometric evaluation in pediatric patients in order to diagnose corneal abnormalities earlier and, ideally, to avoid progression to such advanced stages and subsequent losses in quality of life, this study sought to compare corneal parameters and indices among healthy children, those with ocular allergy, and those with KC using the OCULUS Pentacam Scheimpflug topography system to identify thresholds that can be used to clinically diagnose KC.

## Methods

This cross-sectional study was performed at the University of Campinas (UNICAMP), in Campinas, São Paulo, Brazil. Written informed consent was obtained from all participants’ parents or legal guardians, and the study was approved by the University of Campinas (UNICAMP) ethics committee. The protocol was in compliance with Good Clinical Practices and the tenets of the Declaration of Helsinki (1996).

### Participants

Children with a diagnosis of KC and/or ocular allergy were enrolled during routine visits to the local ophthalmology department. All participants underwent a comprehensive ophthalmological examination, which included a medical history review, best-corrected visual acuity (BCVA), slit-lamp biomicroscopy, autorefraction, and keratometry. KC was diagnosed by experienced cornea specialists based on clinical (including one or more of the following: refractive error, scissoring reflex during retinoscopy, Vogt’s striae, and Fleischer's ring) and topographic criteria (modified Rabinowitz-McDonnell criteria for keratoconus which is based on keratometric readings greater than or equal to 47.2 diopters (D) and an inferior–superior asymmetry (I–S) value greater than or equal to 1.4 D)^28^, which included early and advanced KC. The mild ocular allergy diagnosis was based on a medical history of allergic conjunctivitis and ocular pruritus; some severe diseases also had other ocular signs of allergy, such as conjunctival papillae and Horner-Trantas limbal lesions. The exclusion criteria included inability to undergo the ocular exam, other ocular diseases (strabismus, corneal scars, amblyopia, cataracts, retinal disorders), trauma, ocular surgery, and contact lens wear.

A cohort of healthy children was enrolled from a public school that was chosen because of its involvement in a hospital partnership program with the government. The exclusion criteria consisted of the inability to undergo the ocular exam, a history of any ocular diseases (including strabismus, corneal scars, amblyopia, cataracts, retinal disorders, ocular allergy), trauma, ocular surgery, contact lens wear, and a topographic diagnosis of corneal ectasia based on the modified Rabinowitz-McDonnell criteria for keratoconus (see above)^28^. Finally, informed consent was provided by parents or legal guardians.

### Examination

The ocular tomographic exam was performed using the OCULUS Pentacam Scheimpflug topography system according to manufacturer’s instructions. The topographic, pachymetric, and elevation parameters of the images captured were used to create maps, graphs, and indices to be evaluated and compared between the groups. Only eyes that had acceptable image quality were included. If both eyes of a given control subject met the eligibility criteria, the right eye was included; when a given KC patient’s image quality was acceptable for both eyes, the eye with the more advanced case of KC was included.

### Statistical analysis

For the descriptive analyses, categorical variables were presented as absolute and relative frequencies, while continuous variables were summarized using means and standard deviation (SD). The effect of potential risk factors was evaluated via univariate logistic regression analysis. The models compared the effect of each variable in paired comparisons of the model groups: healthy controls versus KC patients, healthy controls versus ocular allergy patients, and KC patients versus ocular allergy patients. Since the diagnosis groups differed in terms of sex and age, the multivariate logistic models used were adjusted for the potential confounding factors of those two variables. Finally, the area under the receiver operating characteristic (ROC) curves (AUCs) was used to assess and compare the ability of each variable to discriminate between the eyes in each group, and the optimal diagnostic cutoff was estimated using the method provided by Liu^29^.

All statistical analyses were performed using the commercially available software Stata, version 16 (StataCorp LP, College Station, TX). The alpha level (type I error) was set at 0.05.

## Results

One hundred and eighty-two eyes of 182 children were included in this study. Out of these, 99 (54%) were classified as healthy or normal, 32 (18%) were classified as having as ocular allergy, and 51 (28%) were determined to have KC. Most subjects (69%) were male. Mean subject age was 9.4 ± 1.3 years old.

We found statistically significant differences among all groups in all of the topometric, pachymetric, and relational thickness indices studied. Table 1 summarizes the demographic and clinical characteristics of the eyes included in the study according to their diagnostic group.

**Table 1:** Demographics and corneal parameters from the OCULUS Pentacam for healthy, ocular allergy, and keratoconus groups.

	Healthy	Allergy	Keratoconus	*P* value*
Age (years)	9.72 ± 0.66	9.78 ± 1.06	10.10 ± 1.27	*p* = 0.138
K1 (D)	42.82 ± 1.23	42.47 ± 1.33	48.06 ± 5.59	*p* < 0.001
K2 (D)	43.70 ± 1.31	43.79 ± 1.49	53.35 ± 5.73	*p* < 0.001
Kmax (D)	44.19 ± 1.33	44.58 ± 1.62	59.29 ± 8.10	*p* < 0.001
astig (D)	0.88 ± 0.49	1.32 ± 0.86	5.29 ± 2.41	*p* < 0.001
q-value	− 0.38 ± 0.16	− 0.45 ± 0.13	− 1.31 ± 0.59	*p* < 0.001
CCT (Μm)	556.02 ± 32.92	542.41 ± 27.01	479.81 ± 43.59	*p* < 0.001
TP (Μm)	550.46 ± 33.16	535.34 ± 26.20	460.77 ± 51.91	*p* < 0.001
ACD (mm)	3.11 ± 0.25	3.62 ± 0.26	3.76 ± 0.41	*p* < 0.001
BAD-D	0.80 ± 0.61	1.12 ± 0.55	9.29 ± 5.70	*p* < 0.001
ARTmax	446.42 ± 75.23	419.10 ± 80.91	189.45 ± 84.36	*p* < 0.001
PPIave	1.00 ± 0.13	0.99 ± 0.14	2.16 ± 1.06	*p* < 0.001
AETP (Μm)	3.23 ± 1.57	3.71 ± 1.81	24.74 ± 14.37	*p* < 0.001
PETP (Μm)	5.29 ± 2.95	6.22 ± 3.27	49.16 ± 29.74	*p* < 0.001
PE (Μm)	10.80 ± 6.08	9.32 ± 3.72	59.26 ± 28.73	*p* < 0.001

*K1* flat keratometry, *K2* steep keratometry *K*_*max*_ maximum keratometry, *Astig* astigmatism in Sim K, *CCT* central corneal thickness, *TP* thinnest pachymetry, *ACD* anterior chamber depth, *BAD-D* Belin/Ambrosio enhanced ectasia display, *ARTmax* Ambrosio’s relational thickness maximum, *PPIave* average pachymetric progression index, *AETP* anterior elevation at the thinnest point, *PETP* posterior elevation at the thinnest point, *PE* maximum posterior elevation.

*Kruskal–Wallis test.

As detailed in Table 2 the distribution of the clinical parameters of the ocular allergy group was compared to those of the KC group. There were statistically significant differences in the index of vertical asymmetry (IVA), keratoconus index (KI), central keratoconus index (CKI), index of height asymmetry (IHA), and index of height decentration (IHD) values (*p* < 0.001), as well as in pupil diameter (*p* = 0.014).

**Table 2:** Comparison of corneal parameters of pediatric ocular allergy patients to those of pediatric keratoconus patients.

	Allergy	Keratoconus	*P* value*
ISV	23.02 ± 6.82	85.10 ± 41.38	*p* < 0.001
IVA	0.18 ± 0.08	0.64 ± 0.38	*p* < 0.001
KI	1.02 ± 0.02	1.51 ± 1.71	*p* < 0.001
CKI	1.01 ± 0.01	1.09 ± 0.06	*p* < 0.001
IHA	7.25 ± 8.64	29.64 ± 22.74	*p* < 0.001
IHD	0.02 ± 0.01	0.09 ± 0.07	*p* < 0.001
DENSITY	16.92 ± 3.20	16.79 ± 2.53	*p* = 0.981
PUPIL (mm)	3.43 ± 0.57	3.13 ± 0.47	*p* = 0.159

Data expressed as mean ± standard deviation.

*ISV* index of surface variation, *IVA* index of vertical asymmetry, *KI* keratoconus index, *CKI* central keratoconus index, *IHA* index of height asymmetry, *IHD* index of height decentration, *Density* optical corneal density, *Pupil* pupil size in mesopic conditions.

*Wilcoxon rank-sum test.

Healthy children differed significantly from KC children in all parameters. The subjects with ocular allergy differed significantly from KC children in terms of all parameters except for anterior chamber depth (ACD). When children with ocular allergy were compared to the controls, the children with ocular allergy had higher astigmatism values and Belin/Ambrosio enhanced ectasia display (BAD-D) readings than the controls, as well as lower pachymetric readings, q-values, and Ambrosio’s relational thickness maximum (ARTmax) values.

**Figure 1:**
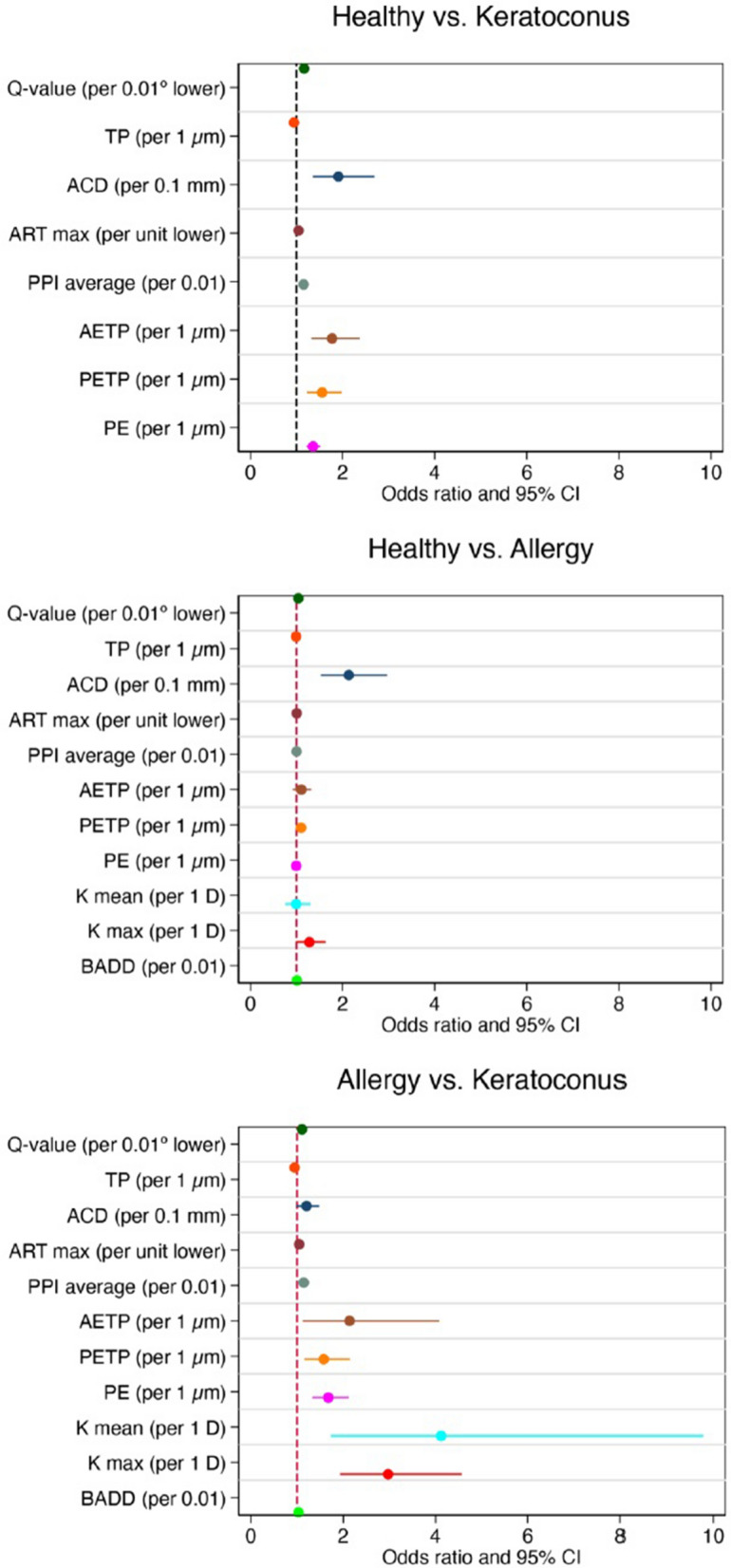
Forest plots for the odds ratio (OR) values and 95% confidence intervals from univariate models comparing (**A**) healthy controls to keratoconus patients, (**B**) healthy controls to ocular allergy patients, and (**C**) ocular allergy patients to keratoconus patients. Dashed vertical line represents an OR of 1. *K1* flat keratometry, *K2* steep keratometry, *Kmax* maximum keratometry, *Astig* astigmatism in Sim K, *TP* thinnest pachymetry, *ACD* anterior chamber depth, *BAD-D* Belin/Ambrosio’s Enhanced Ectasia Display, *ARTmax* Ambrosio’s relational thickness maximum, *PPIave* average pachymetric progression index, *AETP* anterior elevation at the thinnest point, *PETP* posterior elevation at the thinnest point, *PE* maximum posterior elevation.

The calculation of the odds ratio (OR) revealed certain parameters that could easily be used to differentiate between the groups. Results of multivariate analysis that were adjusted for potentially confounding variables (i.e., sex and age) are described in Table 3. Figure 1 shows forest plots that illustrate the estimated increase in the OR of each diagnosis group per unit increase or decrease in the value of each topographical parameter of interest. Maximum keratometry (Kmax) differentiates between groups perfectly because it served as an exclusion criterion for both the control group and ocular allergy group and was therefore not considered in this analysis. The keratometry readings, thinnest pachymetry (TP) values, ACD, q-values, ARTmax, average pachymetric progression index (PPIave), and BAD-D values (BAD-D > 2.42 SD was found to predict the diagnosis group perfectly) were the most reliable parameters for differentiating between normal and KC children. ACD and q-value were found to be the most reliable parameters for differentiating between healthy children and children with ocular allergy. Finally, keratometry readings, BAD-D, q-values, TP, ARTmax, and PPIave were determined to be the most reliable parameters for differentiating between children with ocular allergy and those with KC.

**Table 3:** Multivariate logistic regression models applied to topographical parameters adjusted for age and sex in comparisons between pediatric keratoconus patients, pediatric ocular allergy patients, and healthy controls.

	Controls versus keratoconus		Controls versus allergy		Allergy versus keratoconus	
	OR (95%CI)	*p*	OR (95%CI)	*p*	OR (95%CI)	*p*
K mean (D)	7.19 (2.61–19.80)	**< 0.001**	0.99 (0.75–1.30)	0.78	4.12 (1.74–9.79)	**< 0.001**
Kmax (D)	*	*	1.28 (1–1.63)	0.95	2.97 (1.94–4.57)	**< 0.001**
BAD-D	^a^	^a^	1.01 (1–1.01)	**0.05**	1.03 (1.01–1.05)	**0.003**
q-value	1.16 (1.08–1.25)	**< 0.001**	1.04 (1.01–1.07)	**0.003**	1.11 (1.04–1.17)	**< 0.001**
TP (Μm)	0.94 (0.92–0.95)	**< 0.001**	0.99 (0.98–1)	**0.04**	0.95 (0.93–0.97)	**< 0.001**
ACD (mm)	1.91 (1.35–2.69)	**< 0.001**	2.13 (1.53–2.97)	**< 0.001**	1.20 (0.98–1.48)	0.074
ARTmax	1.04 (1.02–1.05)	**< 0.001**	1 (1–1.01)	0.05	1.04 (1.02–1.07)	**< 0.001**
PPIave	1.15 (1.08–1.23)	**< 0.001**	1 (0.98–1.03)	0.96	1.14 (1.05–1.24)	**0.001**
AETP	1.77 (1.33–2.37)	**< 0.001**	1.1 (0.92–1.32)	0.29	2.14 (1.12–4.07)	**0.020**
PETP	1.56 (1.22–1.98)	**< 0.001**	1.1 (1–1.21)	0.06	1.58 (1.16–2.15)	**0.004**
PE	1.36 (1.21–1.52)	**< 0.001**	0.99 (0.95–1.04)	0.78	1.68 (1.33–2.12)	**< 0.001**

Boldface indicates statistical significance (*p* < 0.05).

*K1* flat keratometry, *K2* steep keratometry, *Kmax* maximum keratometry, *Astig* astigmatism in Sim K, *TP* thinnest pachymetry, *ACD* anterior chamber depth, *BAD-D* Belin/Ambrosio enhanced ectasia display, *ARTmax* Ambrosio’s relational thickness maximum, *PPIave* average pachymetric progression index, *AETP* anterior elevation at the thinnest point, *PETP* posterior elevation at the thinnest point, *PE* maximum posterior elevation.

*K maximum > 47.1 predicts data perfectly.

^a^BAD-D > 2.42 predicts data perfectly.

The AUC was found to be above 0.975 for certain parameters. In these cases, the result implies high sensitivity and specificity when differentiating between healthy children and KC patients, as well as between ocular allergy patients and KC patients. 

**Figure 2:**
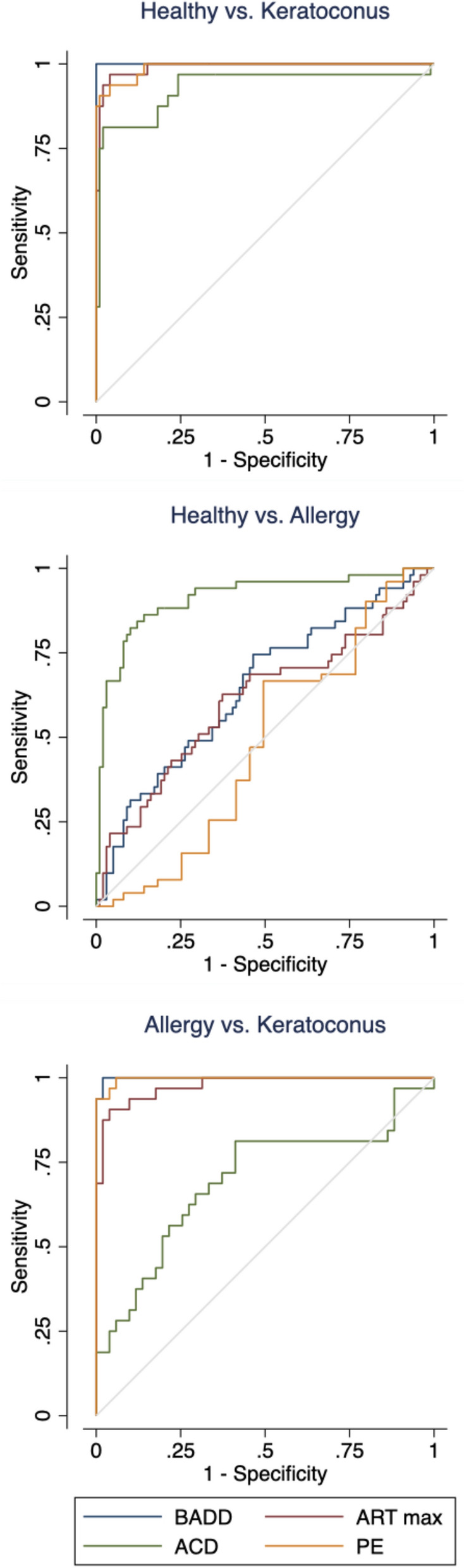
Combined receiver operator curves for BAD-D, ARTmax, ACD, and PE in comparisons between controls and keratoconus patients, controls and ocular allergy patients, and ocular allergy and keratoconus patients.

Figure 2 displays the ROC curve for these parameters. Kmean, Kmax, BAD-D, q-value, ACD, ARTmax, anterior elevation at the thinnest point (AETP), and posterior elevation at the thinnest point (PETP) were found to have an AUC greater than 0.975 in the comparison between healthy controls and KC patients. In the comparison between ocular allergy and KC patients, Kmax, BAD-D, TP, ARTmax, PPIave, and PETP were found to have high AUCs (> 0.975). The BAD-D value was found to be most reliable parameter to differentiate between healthy controls and KC patients and between ocular allergy and KC, with AUC of 1.000 and 0.998, respectively. In the comparison between the control group and the ocular allergy group, the parameter with the highest AUC was found to be ACD (0.913). The AUCs were used to determine cut-off values with the highest sensitivity and specificity for differentiating between the groups. These results are summarized in Tables 4, 5, and 6.

**Table 4:** Area under the receiving operator curve for topographical parameters in a comparison between healthy controls and pediatric keratoconus patients.

	Healthy controls vs. keratoconus patients			
	AUC	Cutoff	Sensitivity (%)	Specificity (%)
K mean (D)	0.976	45.0	94	95
Kmax (D)	*	*		
BAD-D	1.000^a^	2.42^a^	100	100
q-value	0.976	− 0.62	94	98
CCT (Μm)	0.925	527	21	27
TP (Μm)	0.931	506	28	15
ACD (mm)	0.990	3.5	81	97
ARTmax	0.990	334	97	96
PPIave	0.969	1.25	93	95
AETP	0.993	6	96	97
PETP	0.991	11	96	95

*Kmean* mean keratometry, *Kmax* maximum keratometry, *Astig* astigmatism, *CCT* central corneal thickness, *TP* thinnest pachymetry, *ACD* anterior chamber depth, *BAD-D* Belin/Ambrosio enhanced ectasia display, *ARTmax* Ambrosio’s relational thickness maximum, *PPIave* average pachymetric progression index, *AETP* anterior elevation at the thinnest point, *PETP* posterior elevation at the thinnest point, *PE* maximum posterior elevation.

*Kmax > 47.1 predicts the data perfectly and was used as a diagnostic criterion for keratoconus.

^a^BAD-D > 2.42 predicts the data perfectly.

**Table 5:** Area under the receiving operator curve for topographical parameters in a comparison between healthy controls and pediatric ocular allergy patients.

	Healthy controls versus ocular allergy patients			
	AUC	Cutoff	Sensitivity (%)	Specificity (%)
K mean (D)	0.501	43.45	51	56
Kmax (D)	0.601	45.3	43	83
BAD-D	0.648	0.71	74	53
q-value	0.580	− 0.41	59	54
CCT (Μm)	0.593	534	62	34
TP (Μm)	0.407	526	61	34
ACD (mm)	0.913	3.34	84	88
ARTmax	0.611	425	63	63
PPIave	0.496	0.99	51	56
AETP	0.573	3	53	58
PETP	0.608	5	55	64

*Kmean* mean keratometry, *Kmax* maximum keratometry, *Astig* astigmatism, *CCT* central corneal thickness, *TP* thinnest pachymetry, *ACD* anterior chamber depth, *BAD-D* Belin/Ambrosio enhanced ectasia display, *ARTmax* Ambrosio’s relational thickness maximum, *PPIave* average pachymetric progression index, *AETP* anterior elevation at the thinnest point, *PETP* posterior elevation at the thinnest point, *PE* maximum posterior elevation.

**Table 6:** Area under the receiving operator curve (AUC) for topographical parameters in a comparison between pediatric ocular allergy patients and pediatric keratoconus patients.

	Ocular allergy patients versus keratoconus patients			
	AUC	Cutoff	Sensitivity (%)	Specificity (%)
K mean (D)	0.967	45.05	93	94
Kmax (D)	0.996	47.2	100	94
BAD-D	0.998	2.25	100	98
q-value	0.956	− 0.69	90	98
CCT (Μm)	0.908	527	22	29
TP (Μm)	0.991	503	28	15
ACD (mm)	0.699	3.58	81	59
ARTmax	0.977	302	90	96
PPIave	0.976	1.23	93	96
AETP	0.969	7	90	100
PETP	0.989	12	97	98

*Kmean* mean keratometry, *Kmax* maximum keratometry, *Astig* astigmatism, *CCT* central corneal thickness, *TP* thinnest pachymetry, *ACD* anterior chamber depth, *BAD-D* Belin/Ambrosio enhanced ectasia display, *ARTmax* Ambrosio’s relational thickness maximum, *PPIave* average pachymetric progression index, *AETP* anterior elevation at the thinnest point, *PETP* posterior elevation at the thinnest point, *PE* maximum posterior elevation.

## Discussion

In this study, we calculated the diagnostic ability of different corneal parameters using the OCULUS Pentacam Scheimpflug topography system and estimated cutoffs with high sensitivity and specificity for differentiating children with ocular allergy and children with KC from healthy children. To the best of our knowledge, this is the first study to provide normative values for corneal parameters using an age-matched cohort of healthy children. This information may guide better clinical decisions than the cutoffs currently used, which are based on adult populations, and may thus allow for an earlier diagnosis of corneal ectasia.

All parameters studied differed significantly between healthy controls and KC patients. Kmean (45.0 D), BAD-D (2.42), ACD (3.5 mm), ARTmax (334), AETP (6 µm), and PETP (11 µm) were found to be the most reliable parameters in that they offered well-defined cutoffs for differentiating between healthy controls and cases of KC; these parameters also yielded high sensitivity and specificity (all were found to have AUCs above 0.975). This information shows that the Pentacam can easily identify KC in children (similar to its use on adult populations), and that these parameters are the best tools for use in clinical practice.

When ocular allergy parameters were compared to KC parameters, the results also differed significantly. The best parameters (and their cutoffs) were found to be ARTmax (302), AETP (7 µm), and PETP (12 µm), all of which exhibited AUCs above 0.975. These findings support the use of the Pentacam to identify KC in children with ocular allergy. This type of diagnosis and differentiation is important, since children with ocular allergy face a substantial risk of developing KC. These findings also imply that children with ocular allergy should receive an Scheimpflug topography exam using these cutoffs.

In attempts to distinguish between healthy eyes and eyes with ocular allergy, all of the parameters exhibited suboptimal performance (AUC below 0.975). The most reliable parameter was ACD, with an AUC of 0.913, sensitivity of 84%, and specificity of 88% at 3.34 mm of depth. These numbers imply that the parameter is useful, but ACD alone cannot be used to diagnose ocular allergy children. This finding suggests that healthy eyes are similar to eyes with ocular allergy (since only ACD and q-values differed at a *p* < 0.001), and that the Pentacam is not a good tool for identifying this condition in children. This could be explained by the fact that children with ocular allergy without opacities do not have structural damage to the cornea.

Our findings showed that BAD-D was the best tool in the diagnosis of keratoconus (comparing with healthy controls), which is congruent with other studies in adults^30–32^. This parameter is calculated based on a linear regression analysis and derives from different indices considering anterior and posterior elevation and the distribution of corneal thickness: Df (deviation of the normality of the front elevation), Db (deviation of normality of the back elevation), Dt (deviation of normality of corneal thinnest point), Da (deviation of normality of Ambrósio relational thickness), Dp (deviation of normality in average pachymetric progression), Dy (displacement of thinnest point along the vertical meridian), anterior elevation at the thinnest point, posterior elevation at the thinnest point, and Kmax^12^. In the Pentacam display system, each parameter is indicated in yellow (suspicious) if it is ≥ 1.6 SD from the mean or in red (abnormal) if it is ≥ 2.6 SD from the mean^12^, which is very similar to our findings that showed a BAD-D > 2.42 with AUC 1.000 to distinguish healthy children from KC and a BAD-D > 2.25 with AUC 0.998 to distinguish OA children from KC. Different studies in adults showed similar values between healthy controls and clinical keratoconus, ranging from 1.83 to 2.615 (most of all > 2.00 SD)^32–38^. Lower values were found in studies using healthy controls and subclinical keratoconus, ranging from 1.22 to 1.61 SD^32,33,36,37,39,40^, which is different from our study since we included patients with frank KC. Subclinical keratoconus is widely defined as a topographically normal eye that has frank KC in the fellow eye, or subtle topographic changes without clinical signs of KC or a change in visual acuity^41–43^. This information suggests that lower values used for adults could aid in screening for subclinical KC in children, but more studies are needed to confirm this hypothesis.

This information should help clinically to use Pentacam to diagnose KC in children with important risk factor for development of the disease, that is ocular pruritus. It can identify a normal cornea (that is similar to the cornea of OA cornea) in children and distinguish from KC children (in early and advanced cases). This should help to quickly diagnose KC in all children (normal and with OA) and to find early stages of the disease, preventing loss of quality of life and evolution to more advanced cases, since we can treat much earlier. For this essential task, BAD-D can play an important role, since it could easily distinguish between healthy and KC children, and between OA and KC children.

Previous studies have provided substantial information and many normative values for corneal power, astigmatism, corneal thickness, and pachymetric progression indices in adults and healthy children^13^. However, such parameters have not been studied in children with keratoconus or ocular allergy using current tomographers, such as the OCULUS Pentacam Scheimpflug topography system. This is the first study to describe corneal parameters in children with ocular allergy and keratoconus using the Pentacam, as well as the first to compare healthy children, children with ocular allergy, and children with keratoconus, providing us the information we needed for the diagnose of KC in children in early and advanced cases.

The parameters found in normal children were very similar to what was found for adults in literature^44^, which suggests that their corneas are similar too and that the parameters that we use clinically for adults should be very similar for the children, but other studies are needed to confirm this.

This study has certain limitations. Subject age ranged from 7 to 11 years, and it is not known how the corneas of children outside of this age bracket behave or whether these same parameters can be applied to other pediatric age groups. Though the occurrence of KC is more common in adolescents, it is also important to note that, while the tomographic exam takes only two seconds to capture all of the images, the patient must remain immobile with eyes fixated on the target, conditions which could be difficult for younger patients to comply with. Children with ocular allergy can be even more challenging to image because of the inflammation and photosensitivity found in some patients with symptomatic disease; furthermore, severe disease causes corneal opacities, and images therefore cannot be captured with high quality of images using a Scheimpflug topography system.

## Conclusion

This is the first study to identify tomographic parameters of the cornea that can be used to distinguish between healthy children, children with ocular allergy, and children with keratoconus. Keratometry indices, q-value, ACD, ARTmax, BAD-D, AETP, and PETP were found to be the most reliable parameters for differentiating between healthy eyes and cases of keratoconus. TP, ARTmax, and PPIave were found to be the most reliable parameters for differentiating between ocular allergy cases and cases of keratoconus. Finally, none of the parameters alone were found to be reliable for distinguishing between healthy eyes and cases of ocular allergy in children. This should help to identify early diseases in children and in children with risk factor for keratoconus.
